# Developing a Core Outcome Set and a Core Outcome Measurement Set for Studies Evaluating Interventions to Minimize Physical Restraint Use in Adult Intensive Care Units: Protocol for a Modified Delphi Study

**DOI:** 10.2196/76405

**Published:** 2025-11-03

**Authors:** Ziad Alostaz, Wendy L Ellis, Sangeeta Mehta, Mohammed Aldalaykeh, Nasrin Alostaz, Raajpreet Sekhon, Abeer Omar, Craig Dale, Louise Rose

**Affiliations:** 1 Faculty of Health Sciences George Brown College Toronto, ON Canada; 2 Interdepartmental Division of Critical Care Medicine University of Toronto Toronto, ON Canada; 3 College of Nursing, QU-Health Sector Qatar University Doha Qatar; 4 Faculty of Health Disciplines Athabasca University Alberta Canada; 5 Fleming School of Nursing Trent University Peterborough, ON Canada; 6 Lawrence S. Bloomberg Faculty of Nursing University of Toronto Toronto, ON Canada; 7 Florence Nightingale Faculty of Nursing King's College London London United Kingdom

**Keywords:** core outcome set, core outcome measurement set, Delphi process, physical restraint minimization, intensive care unit, ICU

## Abstract

**Background:**

Heterogeneity in outcome selection and measurement methods has been noted in studies examining the minimization of physical restraint use in adult intensive care units (ICUs). This variability undermines evidence synthesis, limiting the development of evidence-based approaches to minimize restraint use and improve patient outcomes.

**Objective:**

This protocol outlines the methods for developing international consensus on core outcomes and standardized measurement approaches for studies focused on physical restraint minimization in adult ICUs.

**Methods:**

We will follow the Core Outcome Measures in Effectiveness Trials Handbook. Drawing on our previous work, including a scoping review of studies on physical restraint minimization and interviews with family members, we will compile a list of potential outcomes for a 2-round Delphi survey. In round 1, stakeholders will rank outcomes using the GRADE (Grading of Recommendations, Assessment, Development, and Evaluations) scale. In round 2, they will review the aggregated results for rescoring and refinement. A consensus meeting using the modified nominal group technique will finalize the core outcome set, followed by another meeting to agree on standardized measurement methods.

**Results:**

Research ethics board approval is in progress. Recruitment has not yet begun. Project initiation is anticipated in January 2026, with completion planned for April 2027 and publication of findings is expected by June 2027.

**Conclusions:**

This study will be the first to establish a core outcome set and a core outcome measurement set for minimizing restraint use in adult ICUs. Standardization will enhance comparability across future studies and support evidence synthesis. The resulting outcome and measurement sets will provide a foundation for high-quality research and guide evidence-based strategies to improve patient safety and care in ICU settings.

**Trial Registration:**

COMET Initiative 3368; https://tinyurl.com/yubfzre9

**International Registered Report Identifier (IRRID):**

PRR1-10.2196/76405

## Introduction

### Background

Physical restraints are frequently used in intensive care units (ICUs) to prevent agitated patients from interfering with life-saving devices such as endotracheal tubes [1,2]. However, emerging evidence indicates that restraints may not prevent device removal in patients who are critically ill and could lead to a range of adverse psychological consequences for patients [3]. These include an increased risk of developing or worsening preexisting delirium or agitation and long-term effects such as a higher risk of posttraumatic stress disorder after ICU discharge [4,5]. The adverse impact of physical restraints extends beyond the patients themselves; family members frequently report feelings of helplessness and profound emotional responses such as sadness and anxiety [6,7]. ICU clinicians also experience emotional uncertainty and moral conflict when making decisions about restraint use as they navigate the complex balance between perceived risks and benefits [8,9]. Considering these adverse outcomes and the limited evidence supporting the effectiveness of physical restraints in preventing device removal, many international guidelines recommend minimizing their use in the ICU and applying restraints only as a last resort after exhausting all potential alternatives [10-12].

With the growing recognition of the importance of restraint minimization in adult ICUs, studies evaluating interventions to reduce physical restraint use continue to proliferate. A critical gap in the current body of evidence on physical restraint use and minimization is the absence of a systematic approach to selecting, measuring, and reporting outcomes [13]. This lack of standardization prevents the pooling of studies in systematic reviews and meta-analyses, which is critical for determining true effect sizes and generating robust guidance to inform practice guidelines [14]. In the absence of such evidence-based guidance, restraint practices may become inconsistent, potentially leading to negative patient outcomes such as unplanned extubations, physical harm, and psychological distress [15]. Furthermore, outcome selection in existing literature on physical restraint use and minimization is often driven by researchers’ perspectives, potentially overlooking outcomes that hold greater significance for patients and their families [13].

To address these gaps in the variability of outcome selection and measurement, the development of a core outcome set (COS) and a core outcome measurement set (COMS) is crucial. A COS is a standardized list of outcomes that are reached through consensus as essential to report across all trials within a specific health condition or intervention area [16]. A COMS is a set of standardized and agreed-upon measurement tools for assessing the outcomes identified in the COS [17]. Currently, the absence of standardization limits the ability to compare results across studies, contributes to research waste, and undermines the translation of evidence into practice. Guided by the principle that standardized outcomes and measurements enhance comparability and ensure alignment with stakeholder priorities, a COS and a COMS promote consistency, improve the quality of systematic reviews and meta-analyses, and facilitate clearer guidance for practice and policy [18]. Importantly, by involving patients, families, and clinicians in determining which outcomes matter most, a COS ensures that the research agenda is aligned with the priorities of those directly affected by restraint practices [19,20]. This protocol outlines the methods we will use to establish an international consensus on the core outcomes to be prioritized and reported as well as the standardized methods for measuring these outcomes in studies aimed at minimizing physical restraint use in adult ICUs.

### Scope

#### Health Condition and Target Population

The focus of this COS and COMS is on adult patients who are subjected to physical restraint use during their ICU stay. In addition, this COS focuses on individuals at risk of physical restraint use, specifically those with an endotracheal tube. This population includes patients with various medical conditions for whom physical restraints may be used to promote safety or prevent device removal.

#### Intervention

This COS and COMS include any nonpharmacological intervention aimed at minimizing physical restraint use in adult ICU patients.

#### Context of Use

This COS and COMS are intended for use in adult ICUs across diverse health care settings, including academic medical centers and community hospitals. Although the COS and COMS to be developed are intended for use in research, they can also be applied in clinical audits and quality improvement initiatives aimed at evaluating interventions to minimize physical restraint use in adult ICUs; for instance, hospitals may use the COS to standardize outcome reporting during audit cycles evaluating physical restraint minimization interventions, allowing for benchmarking across units or regions. The COS and COMS are applicable regardless of regional practices or institutional protocols, promoting consistency and comparability in outcome reporting.

## Methods

### Overview

We have developed this protocol in alignment with the Core Outcome Set–Standardized Protocol statement (Multimedia Appendix 1) and have adhered to the guidelines outlined in the Core Outcome Measures in Effectiveness Trials (COMET) Handbook (version 1.0) [21,22]. This protocol has been registered in the COMET database (3368). Key stakeholders, including family members, clinicians, and researchers, provided feedback in separate informal conversations during the development of this protocol, helping refine data collection methods to ensure that they were appropriate, feasible, and respectful.

Building on this foundation and to ensure broad representation, methodological rigor, and feasibility, the study will proceed in three structured stages: (1) preparatory stage and ethics approval, (2) establishing the COS, and (3) development of the COMS. Each stage is described in detail in the following subsections. A comprehensive project timeline, including stages, key activities, and anticipated timeline for completion, is provided in Multimedia Appendix 2.

### Stage 1: Preparatory Stage

Our steering committee will include 3 experts with clinical and research expertise in physical restraint use in the ICU, along with a patient or family representative. As the study progresses, we will expand our steering committee to include representatives from key stakeholders across all participating countries. To guide and optimize our engagement with ICU survivors and families throughout the development and execution of the COS study, we will use the patient and public involvement toolkit and COMET checklist for public research partners and COS study developers involved in designing a COS study [23].

### Stage 2: Establishing the COS

#### Information Sources

##### Outcome List Generation

We will use the following data sources to generate a list of potential outcomes.

###### Scoping Review

We will use our previously conducted scoping review of studies on interventions to minimize physical restraint use in adult ICUs to generate a list of outcomes reported in the included studies [13]. The scoping review followed established methodology, with a search strategy developed iteratively and informed by an experienced information specialist. A combination of controlled vocabulary terms (eg, “restraint, physical,” “intensive care unit,” and “critical care”) and keywords (eg, “restrain*,” “intensive,” “critical,” and “acute”) was used. Seven databases, along with gray literature, were searched from inception to 2019. Two researchers independently screened and extracted data on study characteristics, outcomes, and outcome measurement instruments and definitions. Studies included were those in which the primary goal was to minimize physical restraint use in adult ICUs. Studies published in languages other than English were excluded. We will update our search strategy and execute a search to capture both published studies and ongoing trials from 2019 to 2025, using the same databases and gray literature sources. A table of outcomes, their definitions, and corresponding measurement tools will be created using studies included from the updated search, along with those already included in the previously conducted scoping review. We will calculate the proportion of studies reporting each outcome and rank them by frequency. Measurement tools will be evaluated for their psychometric properties using the COSMIN (Consensus-Based Standards for the Selection of Health Measurement Instruments) checklist [24].

###### Family Member Interviews

We will review the transcripts from our previously conducted interviews with family members regarding their perspectives on physical restraint practices and minimization in adult ICUs [6]. This was a qualitative descriptive study that involved one-on-one semistructured interviews with 15 family members in a medical-surgical trauma ICU in Toronto, Ontario, Canada. The study inclusion criteria were as follows: (1) self-identification as a family member aged 18 years or older who had observed the use of physical restraint on their loved one for at least 6 hours in the ICU, (2) a history of visiting the patient in the ICU during the period when they were physically restrained, and (3) the ability to speak English. The theoretical domains framework was used to guide both data collection and analysis, using a deductive content analysis approach. Three themes emerged: (1) barriers and facilitators to physical restraint minimization; (2) unilateral decision-making regarding physical restraint use, where clinicians made decisions without adequate communication with families or obtaining consent; and (3) the emotional impact of physical restraint use, with families experiencing sadness and shock and believing that patients would have similar emotional responses. We will thoroughly review the interview transcripts and create a comprehensive list of outcomes reported by family members.

##### Outcome List Reduction

Two researchers from the research team will independently review the outcome list derived from the family interviews and merge it with the outcome list obtained from the scoping review, consolidating similar outcomes and eliminating redundancies. The 2 researchers will then independently group outcomes with similar definitions under the same outcome name; for example, “Duration of physical restraint use (hours)” and “Average number of hours per day a patient is restrained” will both be grouped under the outcome name “Duration of restraint use.” The outcomes will then be classified into broader outcome domains in accordance with the COMET taxonomy [25]; for instance, “Duration of restraint use” and “Type of restraint used” will be grouped under the “Clinical outcomes” domain within the COMET taxonomy. Outcomes that do not duplicate those identified in the scoping review will be categorized according to the COMET taxonomy and marked as being identified exclusively by patients and family members. Any disagreements in the process of outcome reduction and categorization will be resolved through discussion or, if necessary, by consulting a third researcher serving as an independent arbiter.

The final outcome list will undergo a thorough review by the steering committee to ensure accurate categorization of outcomes, clarity, and the use of simple language that can be easily understood by all stakeholder groups. Before the Delphi process begins, the Delphi survey will undergo pilot testing with at least 4 researchers or clinicians external to the core research team and 4 patients or family members to assess face validity and ensure that the wording is clear and easily understood. As part of this pilot test, participants will be asked to report the time taken to complete the survey and to provide feedback on any difficulties in maintaining attention or engagement. On the basis of this feedback, we will make all necessary refinements to optimize clarity, usability, and feasibility for all stakeholder groups.

#### Stakeholder Recruitment and Sample Monitoring

##### Overview

We will seek a purposive and maximum variation sample from 3 key stakeholder groups—ICU survivors and their family members, researchers, and clinicians—drawn from an international population [26,27]. ICU survivors and family members will be included to capture patient-centered perspectives that may differ from those of clinicians and researchers. Clinicians and researchers will be recruited for their specialized expertise, ensuring that the selected outcomes are clinically meaningful, measurable, and aligned with current evidence and best practices. Multimedia Appendix 3 outlines the key sampling characteristics for stakeholders. To ensure that our participants represent a diverse mix of these characteristics, we will continuously monitor participant demographics during recruitment and adjust recruitment strategies as needed to allow for maximum variation and address any gaps in representation.

##### Recruitment Methods

To recruit ICU survivors and family members, we will use a multimethod strategy that includes the use of a dedicated study X (formerly Twitter) account, engagement with relevant patient and family support and advocacy groups (eg, the Canadian Critical Care Trials Group Patient & Family Partners Community), outreach through personal contacts, and snowball sampling. Participants will be included if they, or someone they consider a family member, have experienced physical restraint in an ICU setting. In addition, they must be able to read and speak English.

Researchers will include individuals who have published at least one clinical study related to any area of adult critical care. Clinicians will encompass physicians, registered nurses, respiratory therapists, and allied health care professionals (eg, physical therapists) with varying levels of experience in adult ICUs. All participants must be able to read and speak English. Recruitment of both researchers and clinicians will be conducted through personal contacts, snowball sampling, and emails to corresponding authors of the studies included in our scoping review. While practical, this approach may result in over- or underrepresentation of stakeholder groups, potentially influencing which outcomes are prioritized in the Delphi survey. To monitor and mitigate this, we will track participant demographics, professional backgrounds, and geographic distribution across rounds. Broad outreach strategies, including social media campaigns and dissemination through professional networks, will be implemented alongside targeted engagement with patient and family advocacy groups serving diverse communities to enhance representativeness. Recruiting participants who can speak and read only English may exclude non-English-speaking stakeholders, potentially underrepresenting certain cultural or patient perspectives. Although resource constraints preclude translation of study materials, we will actively engage organizations that serve multilingual populations to facilitate participation and ensure diverse perspectives.

##### Sample Size

There are no strict guidelines or formal power calculations for determining the sample size in a COS consensus process, as it is influenced by practical considerations and the scope of the COS [22]. However, it is emphasized that broad representation across all key stakeholder groups is crucial. To ensure comprehensive and inclusive input, we will aim to recruit 60 to 80 participants, with 20 to 30 participants per individual stakeholder group selected deliberately to represent all characteristics outlined in Multimedia Appendix 3. For the consensus meetings, we will aim to recruit 15 to 20 participants, including representatives from each key stakeholder group. This sample size was selected based on published COS studies and the recommendation in the COMET Handbook [22,28,29]. Participants will be purposively selected to ensure diverse perspectives across disciplines, geographic locations, and experience with physical restraint use in ICUs.

#### Consensus Process for the COS

The consensus process will include 2 web-based modified Delphi survey rounds, followed by a modified nominal group meeting.

##### Delphi Survey Round 1

###### Overview

The Delphi survey will be administered using REDCap (Research Electronic Data Capture; Vanderbilt University)*,* a secure platform that allows customized survey design and automated email distribution, making it well suited for multiround Delphi studies. The first part of the survey will collect baseline data to characterize the stakeholder sample and assign each participant a unique identifier. This identifier will allow for personalized follow-ups and reminders in subsequent rounds. Participants will be asked to self-select their stakeholder group (eg, ICU survivors, family members, clinicians, or researchers).

The second part will present the list of outcomes described in lay terms, with any medical terms provided in parentheses, for participants to rate based on importance, without consideration of measurability or feasibility. The importance of each outcome will be rated using the GRADE (Grading of Recommendations, Assessment, Development, and Evaluations) approach on a 9-point Likert scale: 1 to 3 (not important for inclusion), 4 to 6 (important but not critical), and 7 to 9 (critical for inclusion) [30]. The GRADE scale was selected because it is recommended by COMET to facilitate maximum discrimination between survey items [22]. To reduce incomplete responses, the survey will include an “unable to score” option. Participants will also have the opportunity to provide free-text comments and suggest additional outcomes. To minimize presentation bias, the order of outcome domains will be randomized. Participants will have 3 weeks to complete the survey, with 2 reminder emails sent: one at the start of the second week and another at the beginning of the third week.

###### Round 1 Analysis

For each outcome, we will calculate the proportion of participants rating it as 7 to 9 (critical for inclusion), 4 to 6 (important but not critical), or 1 to 3 (not important for inclusion) for the entire stakeholder panel and each stakeholder group separately. All survey responses will be analyzed on an item-by-item basis. Partial completions will be retained, as responses to individual items may still offer valuable insights and contribute meaningfully to the consensus process.

If new outcomes emerge in round 1, the steering committee will carefully review them to ensure that they represent distinct concepts (meaning that they are not overlapping in definition or intent with existing outcomes), are clearly articulated, and are free from duplication before being included in round 2. If needed, a third Delphi survey round may be conducted specifically for these new outcomes, allowing for 2 rounds of scoring for each outcome before the final consensus meeting.

##### Delphi Survey Round 2

###### Overview

Participants who complete round 1 will be invited to take part in round 2. All outcomes from round 1 will be carried forward to round 2. Any newly identified outcomes from round 1 will also be introduced in round 2 for scoring on the 9-point GRADE Likert scale. Participants will receive a summary of responses from round 1, including aggregated feedback from all stakeholder groups, a summary of responses for each stakeholder group, and their own individual responses. Participants will be asked to rescore each outcome using the GRADE scale. If a participant adjusts their rating, moving it into a new category (eg, from “important but not critical” to “critical for inclusion”), they will be prompted to provide a free-text rationale for the change. The same 3-week response window and 2 email reminders at the beginning of weeks 2 and 3 will be sent to all participants.

###### Round 2 Analysis

For round 2, we will calculate the proportion of participants rating each outcome as either “critical for inclusion” or “not important for inclusion.” As recommended by the COMET Handbook, outcomes will be brought forward to the consensus meeting only if at least 70% of the respondents rate them as “critical for inclusion” and less than 15% rate them as “not important for inclusion” across all stakeholder groups combined [22]. We will also calculate the proportion of participants rating each outcome separately within each stakeholder group. Outcomes with mixed ratings—for example, those that reach consensus in the patient or family group but with clinicians on the fence—will be brought forward to the consensus meeting for in-depth discussion and potential rerating.

##### Attrition Bias Between Rounds

Attrition bias may arise if a subset of participants drops out or fails to respond between rounds, resulting in a nonrepresentative final group [22]. Differential attrition across stakeholder groups (eg, clinicians, researchers, or patient representatives) may disproportionately influence which outcomes are prioritized, potentially skewing the Delphi survey results toward the perspectives of participants who remain engaged. To mitigate this risk, participants will receive personalized invitations, multiple reminders, and direct access to the research team for support [31]. The survey will be scheduled outside holiday periods, and its length will be optimized to reduce participant burden. A 3-week response window will be provided, and participants will be acknowledged for their contributions through a personalized thank-you email. Regular updates on study progress and findings will also be shared to maintain engagement.

Attrition rates will be monitored and reported in accordance with Core Outcome Set–Standards for Reporting guidance, including the number and characteristics of participants who drop out. Characteristics of completers and noncompleters will be compared to assess whether systematic differences exist. Although there is no strict threshold for an acceptable response rate, an attrition rate of up to 20% is generally considered acceptable [22]. If attrition exceeds this threshold between rounds 1 and 2, we will extend the survey period and invite additional eligible participants to ensure sufficient representation.

#### Consensus Meetings for the COS

To finalize the outcomes for inclusion in the COS, we will conduct 1 online consensus meeting, offered in 2 separate time slots to accommodate different international time zones and maximize stakeholder participation. If selection of the final outcomes for the COS cannot be decided, an additional meeting will be scheduled. All meetings will be conducted via Zoom (Zoom Video Communications, Inc) and will include broad representation from all key stakeholder groups. The research team will facilitate the meetings, using Zoom features such as breakout rooms for small-group discussions, virtual whiteboards, and chat functions to enhance engagement and streamline the consensus process. The consensus meeting will follow a structured process: (1) project leads provide an overview of Delphi survey round 2 results and the purpose of the meeting; (2) participants engage in small-group discussions to begin ranking outcomes that reached the consensus threshold in Delphi survey round 2; (3) small groups present their rankings in a large-group discussion; (4) participants are reshuffled into new small groups to refine rankings with facilitator guidance; and (5) small groups share revised rankings in a final large-group discussion, followed by formal anonymous voting to confirm consensus on the final COS. This approach ensures balanced input from all stakeholders, minimizes the influence of dominant voices, and allows participants to prioritize outcomes from most to least important.

### Stage 3: Development of the COMS

#### Information Source

For each outcome that achieves consensus in our COS consensus meeting, we will identify the most relevant outcome measurement instruments and their corresponding measurement time horizons from the studies included in our scoping review. If an outcome identified during the consensus meeting was not captured in the scoping review, we will consult existing critical care–specific COSs to determine appropriate measurement instruments and time horizons [29,32,33]. Each identified instrument will be appraised using the COSMIN risk-of-bias checklist to assess measurement properties such as reliability and validity. Our research team includes a member with methodological expertise in outcome measurement, including the application of the COSMIN framework and evaluation of psychometric properties in critical care contexts. We will also assess feasibility through consultations with our research team, relevant stakeholders experienced in using these instruments in ICU settings, and representatives from our patient and family member group. In preparation for the COMS consensus meeting, we will develop and distribute comprehensive “measure cards” to all participating stakeholders, using plain language to ensure that they are understandable to all stakeholder groups. Each card will outline the scope of the COMS, provide a clear description of each proposed measurement instrument, and summarize its psychometric properties and feasibility of use in clinical practice.

#### Consensus Meetings for the COMS

We will send an invitation via email to stakeholders involved in the COS consensus meetings, inviting them to participate in online COMS consensus meetings on Zoom. To accommodate participants from different time zones and encourage maximum involvement, we will provide several scheduling options. We will ensure that attendees represent our key stakeholder groups.

Before the consensus meetings, stakeholders who have agreed to participate will be invited to propose additional measurement instruments for consideration. Any newly suggested instruments will undergo a rigorous evaluation by the research team to determine their validity, reliability, and feasibility before inclusion in the consensus discussions.

Members from the research team will facilitate the COMS consensus meetings. At the outset of these meetings, participants will be provided with a clear overview of the COMS scope, inclusion criteria for measures (ie, reliability, validity, and feasibility), and the voting process. For each outcome, measurement instruments outlined in the briefing documents will be presented, followed by structured small- and large-group discussions. Voting will be conducted using Zoom’s polling function, with response options of yes, no, or uncertain for inclusion in the COMS. Each participant will vote for a single option per poll and will be encouraged to select “uncertain” rather than abstain if undecided. Consensus will be defined as at least 70% of participants voting “yes” for inclusion. If consensus is not reached in the first vote, additional discussion will follow before a second round of voting. If consensus remains unresolved, the reasons will be documented. After finalizing the selection of measurement instruments, participants will discuss and vote on appropriate measurement time horizons for those measures that reached consensus.

### Ethical Considerations

Before study commencement, we will obtain centralized ethics approval through our home institution and secure research ethics board or equivalent approval from any institution or country that requires it. Informed consent will be obtained from stakeholders before the COS and COMS consensus meetings and will be documented via signed forms. Clear information sheets outlining the study purpose, procedures, voluntary participation, and withdrawal rights will be provided. Participation in the Delphi survey rounds will be considered implied consent. All data will be handled with strict confidentiality, encrypted, stored on a password-protected computer, and accessible only to the research team. Data retention and destruction will follow institutional and regulatory requirements, ensuring compliance with applicable privacy regulations. Participants will be offered compensation for their time in the form of a US $20 gift voucher, subject to availability of funding. This will be clearly explained during the consent and recruitment process.

## Results

The outlined methods will support the development of a COS and a COMS for studies on physical restraint minimization in adult ICUs. Establishing these standardized sets could strengthen evidence synthesis, drive meaningful practice change, and ultimately improve patient outcomes. Research ethics board approval is in progress. Recruitment has not yet begun. The project is scheduled to begin in January 2026 and conclude by April 2027, with results expected to be published by June 2027.

## Discussion

This study is expected to produce a consensus-based COS and COMS to guide outcome selection and measurement in research focused on physical restraint minimization in adult ICUs. Previous studies on physical restraint use and minimization have used a variety of outcomes and measurement tools, leading to significant heterogeneity that limits the ability to effectively synthesize evidence and inform clinical practice. The development of a COS and a COMS aims to standardize outcome selection and measurement, incorporating input from diverse stakeholders. A key strength of this COS and COMS protocol is its adherence to the recommendations outlined in the COMET Handbook. The protocol has been developed in accordance with relevant guidelines, including the Core Outcome Set–Standardized Protocol, ensuring methodological rigor. In addition, multiple methods will be used to enhance the comprehensiveness of COS and COMS development, including an updated scoping review, a review of previously conducted interviews with family members, a Delphi process, and consensus meetings. Another significant strength is the novelty of this work—no existing COS or COMS currently address this area, making their development both timely and essential. Furthermore, the inclusion of international stakeholders will enhance the global applicability and relevance of the final COS and COMS.

Limitations include restricting the inclusion of studies in our scoping review to those published in English, as well as recruiting only participants who can read and speak English. This may limit the ability to capture outcomes and measurements that are deemed significant by non-English speakers. Another limitation is that the qualitative interviews being reviewed were conducted solely with family members and were not originally designed to explore their perspectives on outcomes they believe should be selected and measured in physical restraint minimization studies. As a result, the identified outcomes from these interviews may not be comprehensive and may not fully reflect the patients’ perspectives. However, by including both ICU survivors and family members in the steering committee and broader panel, we will ensure that the identified outcomes are not only relevant to their experiences but also capture any additional outcomes they deem significant. The COS and COMS will be shared with all Delphi survey and consensus meeting participants through plain-language summaries and email updates, with optional webinars to explain key findings. Results will be published in peer-reviewed journals and presented at relevant conferences, with deposition in the COMET database to ensure accessibility for researchers. To support translation into practice, the COS and COMS will be disseminated through ICU professional organizations and international networks, including the Canadian Critical Care Society and the Restraint Reduction Network, facilitating integration into practice standards. In conclusion, the outlined methods will facilitate the development of a COS and a COMS for studies on physical restraint minimization in adult ICUs. By standardizing outcome selection and measurement, these sets have the potential to enhance evidence synthesis, inform best practices, and ultimately improve patient care and safety.

## Appendix

Multimedia Appendix 1 Standardized protocol statement.

Multimedia Appendix 2 Study timeline.

Multimedia Appendix 3 Key sampling characteristics for stakeholders.

## Abbreviations

COMET: Core Outcome Measures in Effectiveness Trials
COSMIN: Consensus-Based Standards for the Selection of Health Measurement Instruments
GRADE: Grading of Recommendations, Assessment, Development, and Evaluations
ICU: intensive care unit
REDCap: Research Electronic Data Capture
