# Correction: Developing a Core Outcome Set and a Core Outcome Measurement Set for Studies Evaluating Interventions to Minimize Physical Restraint Use in Adult Intensive Care Units: Protocol for a Modified Delphi Study

**DOI:** 10.2196/88851

**Published:** 2025-12-11

**Authors:** Ziad Alostaz, Wendy L Ellis, Sangeeta Mehta, Mohammed Aldalaykeh, Nasrin Alostaz, Raajpreet Sekhon, Abeer Omar, Craig Dale, Louise Rose

**Affiliations:** 1 Faculty of Health Sciences George Brown College Toronto, ON Canada; 2 Interdepartmental Division of Critical Care Medicine University of Toronto Toronto, ON Canada; 3 College of Nursing QU-Health Sector Qatar University Doha Qatar; 4 Faculty of Health Disciplines Athabasca University Alberta, AB Canada; 5 Fleming School of Nursing Trent University Peterborough, ON Canada; 6 Lawrence S. Bloomberg Faculty of Nursing University of Toronto Toronto, ON Canada; 7 Florence Nightingale Faculty of Nursing King's College London London United Kingdom

In “Developing a Core Outcome Set and a Core Outcome Measurement Set for Studies Evaluating Interventions to Minimize Physical Restraint Use in Adult Intensive Care Units: Protocol for a Modified Delphi Study” [1], the authors noted an error in the affiliation of one of the co-authors.

The affiliation for Craig Dale was incorrectly listed as:

Interdepartmental Division of Critical Care Medicine, University of Toronto, Toronto, ON, Canada

This has been corrected to:

Lawrence S. Bloomberg Faculty of Nursing, University of Toronto, Toronto, Ontario, Canada

The correction will appear in the online version of the paper on the JMIR Publications website, together with the publication of this correction notice. Because this was made after submission to PubMed, PubMed Central, and other full-text repositories, the corrected article has also been resubmitted to those repositories.
