# Supplementary material for: Developing a Core Outcome Set and a Core Outcome Measurement Set for Studies Evaluating Interventions to Minimize Physical Restraint Use in Adult Intensive Care Units: Protocol for a Modified Delphi Study
Source: JMIR Res Protoc. 2025 Nov 3;14:e76405. doi: 10.2196/76405 (PMC12624295; doi:10.2196/76405)
Supplement: Multimedia Appendix 1 [file resprot_v14i1e76405_app1.pdf]

Appendices

Appendix 1: The COS-STAP Statement Checklist

**SECTION/TOPIC**	**ITEM No.**	**CHECKLIST ITEM**	**REPORTED ON PAGE NUMBER**
TITLE/ABSTRACT			
Title	1a	Identify in the title that the paper describes the protocol for the planned development of a COS	P (ii) line 1-3
Abstract	1b	Provide a structured abstract	P (ii) line 4-31
INTRODUCTION			
Background and objectives	2a	Describe the background and explain the rationale for developing the COS, and identify the reasons why a COS is needed and the potential barriers to its implementation	P (1) line 1-41
	2b	Describe the specific objectives with reference to developing a COS	P (2) line 42-43<br>P (2) line 1-2
Scope	3a	Describe the health condition(s) and population(s) that will be covered by the COS	P (2) line 4-9
	3b	Describe the intervention(s) that will be covered by the COS	P (2) line 10-12
	3c	Describe the context of use for which the COS is to be applied	P (2) line 13-22
METHODS			
Stakeholders	4	Describe the stakeholder groups to be involved in the COS development process, the nature of and rationale for their involvement and also how the individuals will be identified; this should cover involvement both as members of the research team and as participants in the study	P (5) line 1-44<br><br>P (6) line 1-6
Information sources	5a	Describe the information sources that will be used to identify the list of outcomes. Outline the methods or reference other protocols/papers	P (3) line 21-41<br>P (4) line 1-20<br>P(8) line 15-32
	5b	Describe how outcomes may be dropped/combined, with reasons	P (4) line 21-44
Consensus process	6	Describe the plans for how the consensus process will be undertaken	P(6) line 7-43<br>P(7) line 1-21<br>P(8) line 33-42<br>P(9) line 1-13
Consensus definition	7a	Describe the consensus definition	P(7) 14-17
	7b	Describe the procedure for determining how outcomes will be added/combined/dropped from consideration during the consensus process	P (6) line 10-43<br>P (7) line 1-11
ANALYSIS			
Outcome scoring/feedback	8	Describe how outcomes will be scored and summarised, describe how participants will receive feedback during the consensus process	P (6) line 31-43<br>P(7) Line 12-21

Missing data	9	Describe how missing data will be handled during the consensus process	P (7) line 22-40
ETHICS and DISSEMINATION			
Ethics approval/informed consent	10	Describe any plans for obtaining research ethics committee/institutional review board approval in relation to the consensus process and describe how informed consent will be obtained (if relevant)	P (3) line 10-19
Dissemination	11	Describe any plans to communicate the results to study participants and COS users, inclusive of methods and timing of dissemination	P (10) line 6-17
ADMINISTRATIVE INFORMATION			
Funders	12	Describe sources of funding, role of funders	P (10) line 22-23
Conflicts of interest	13	Describe any potential conflict	P (10) line 21

From: Kirkham JJ, Gorst S, Altman DG, et al. (2019) Core Outcome Set-STAndardised Protocol Items: the COS-STAP Statement. Trials 20, 116. https://doi.org/10.1186/s13063-019-3230-x
