# Supplementary material for: Developing a Core Outcome Set and a Core Outcome Measurement Set for Studies Evaluating Interventions to Minimize Physical Restraint Use in Adult Intensive Care Units: Protocol for a Modified Delphi Study
Source: JMIR Res Protoc. 2025 Nov 3;14:e76405. doi: 10.2196/76405 (PMC12624295; doi:10.2196/76405)
Supplement: Multimedia Appendix 2 [file resprot_v14i1e76405_app2.pdf]

Appendix 2. Study Timeline

Stages	Activities	Timeline
Stage 1: Preparation and Ethics approval	Submit REB application and obtain institutional approvals.	October 2025- January 2026
Stage 2: Establishing the Core Outcome Set (COS)	Identify and reduce potential outcomes (scoping review and family interviews); recruit stakeholders and monitor sample; conduct two Delphi survey rounds with analysis; consensus meeting to finalize COS.	January 2026-July 2026
Stage3: Developing the Core Outcome Measurement Set (COMS)	Conduct consensus meetings to select measurement instrument for each outcome; evaluate feasibility and psychometric properties.	August 2026- February 2027
Dissemination	Prepare manuscripts, conference presentations, stakeholder summaries, and distribute findings to relevant research and clinical communities.	March 2027- June 2027
