# Supplementary material for: Developing a Core Outcome Set and a Core Outcome Measurement Set for Studies Evaluating Interventions to Minimize Physical Restraint Use in Adult Intensive Care Units: Protocol for a Modified Delphi Study
Source: JMIR Res Protoc. 2025 Nov 3;14:e76405. doi: 10.2196/76405 (PMC12624295; doi:10.2196/76405)
Supplement: Multimedia Appendix 3 [file resprot_v14i1e76405_app3.pdf]

Appendix 3. Stakeholder sampling characteristics

a) ICU patient/family member

- Age, years (≤ 65 and >65)
- Sex (female and male)
- Region of residence (North America, Europe, UK, Middle East and others)

b) Clinicians

- Profession (nurse, nurse practitioner, physician, other allied health care practitioner)
- Clinical experience, years (<5, 5-10, and >10)
- Region of residence (North America, Europe, UK, Middle East and others)

c) Researchers

- Research experience, years (<5, 5-10, and >10)
- Published at least one clinical study related to any area of adult critical care
- Region of residence (North America, Europe, UK, Middle East and others)
